# Supplementary material for: Total enzymatic synthesis of cis-α-irone from a simple carbon source
Source: Nat Commun. 2022 Dec 2;13:7421. doi: 10.1038/s41467-022-35232-2 (PMC9715568; doi:10.1038/s41467-022-35232-2)
Supplement: Supplementary file 1 — Supplementary Information [file 41467_2022_35232_MOESM1_ESM.pdf]

# Supplementary Information

# Total enzymatic synthesis of cis-α-irone from a simple carbon source

Xixian Chen[1*], Rehka T[1], Jérémy Esque[2], Congqiang Zhang[1], Sudha Shukal[1], Chin Chin Lim[1], Leonard Ong[1], Derek Smith[1], Isabelle André [2*]

1. Singapore Institute of Food and Biotechnology Innovation (SIFBI), Agency for Science, Technology and Research (A*STAR), Singapore. 31 Biopolis Way, Level 6 Nanos building, Singapore 138669.

2. Toulouse Biotechnology Institute, TBI, Université de Toulouse, CNRS, INRAE, INSA, Toulouse, France. 135, avenue de Rangueil, F-31077 Toulouse Cedex 04, France.

*To whom correspondence may be addressed.

Email: Xixian_chen@sifbi.a-star.edu.sg; isabelle.andre@insa-toulouse.fr

## Supplementary Figures.

a

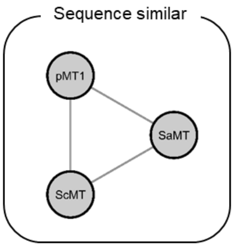


| Variant | Identity (%) | TTN |
|---|---|---|
| pMT1 | 100 | <0.001 |
| SaMT | 68.4 | <0.001 |
| ScMT | 66.32 | ND |

b

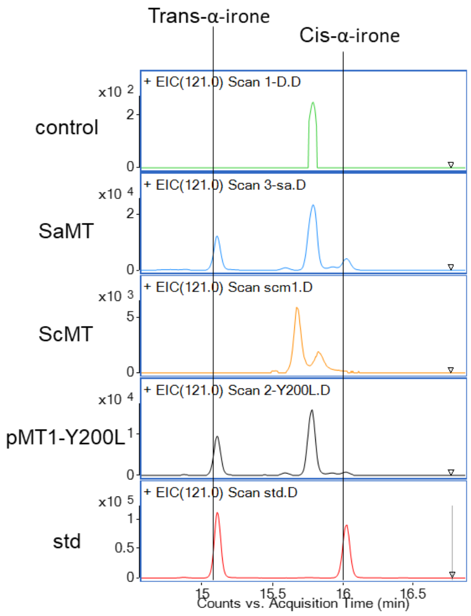


**Supplementary Figure 1.** Identification of the initial methyltransferase activity.

**a**, Selected methyltransferases that are sequence similar to pMT1 were tested for their promiscuous activities to convert psi-ionone to irone. The sequence similarity network showed two methyltransferases (SaMT: NCBI Accession number: ARZ69198.1 and ScMT: NCBI Assession number: WP_003963340) that are more than 60% identical to pMT1, but only SaMT converted psi-ionone to α-irone. **b**, Headspace solid-phase microextraction coupled to gas chromatography- time-of-flight mass spectrometry (HS-SPME-GCMS) analysis of the reaction mixtures containing cell lysates overexpressing IspD (control), SaMT, ScMT or pMT1-Y200L. Methylated products corresponding to trans-α-irone and cis-α-irone were detected in the headspace of reaction by SaMT and pMT1-Y200L. The retention time of the irones are the same as the synthetic chemical standard. The heterologous sequence data are provided in Supplementary data 3.

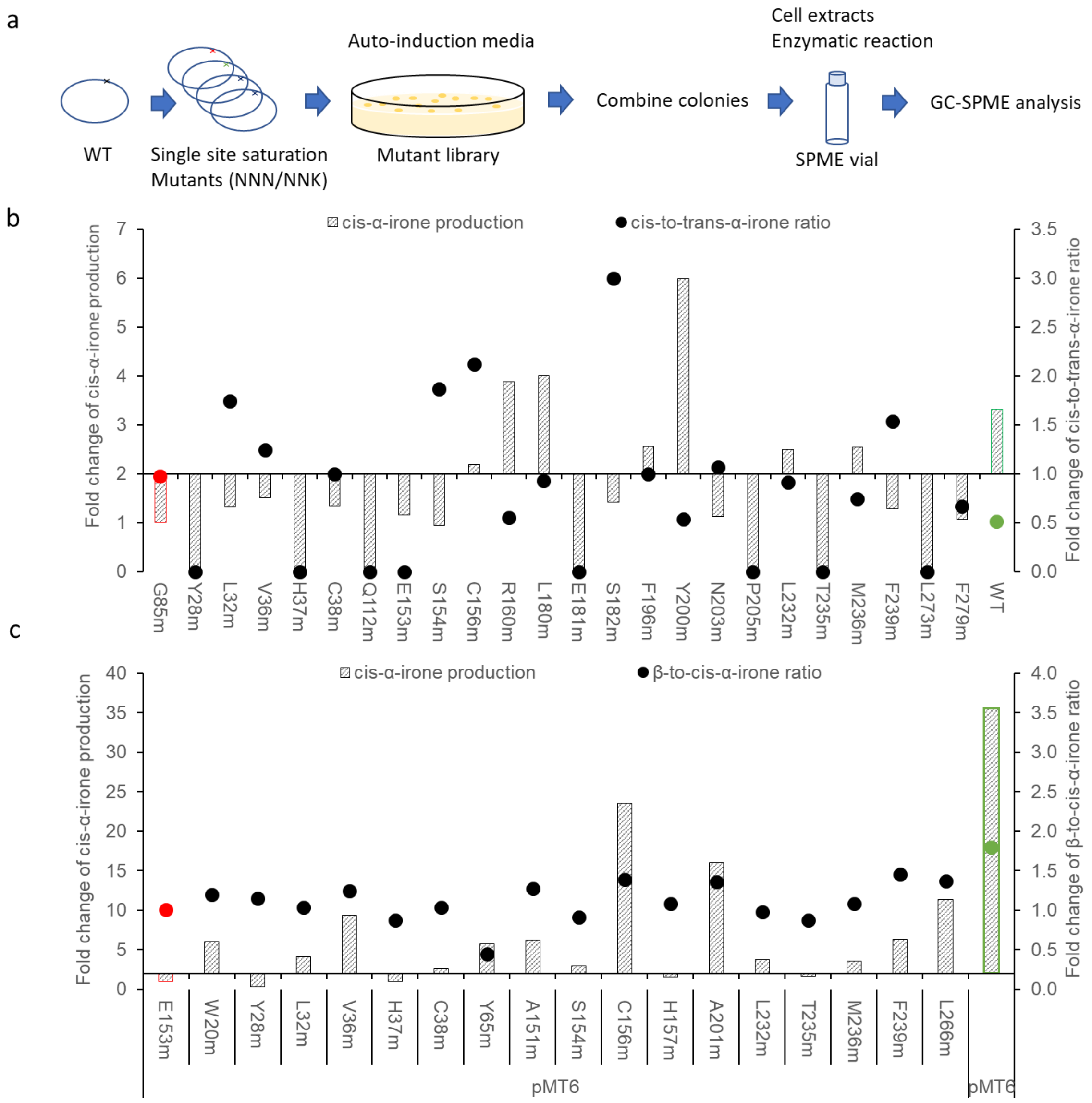


**Supplementary Figure 2.** Site-saturation mutagenesis and pooled-colony reactions to identify the desired residues to mutate.

**a**, Schematic representation of the pooled-colony reactions. One residue was chosen and subjected to site-saturation mutagenesis by using degenerative primers containing NNN or NNK. The plasmid library was then transformed into *E. coli* BL21 (DE3) cells and plated on agar plates containing autoinduction media[1]. Optimization was carried out to ensure that there were more than 200 colonies formed on the agar plates to achieve a good coverage of the mutants. Subsequently, the colonies were collected and resuspended in phosphate saline buffer. Equal number of cells was taken for lysis and cell lysate reactions were carried out in capped SPME-GC vials at 28 $^{o}$C for 2 days. HS-SPME-GCMS analysis was used to quantify the amount of irones produced by each pooled-colony reaction. **b**, Pooled-colony reaction to identify the active site residue(s) to mutate to improve pMT1 activity and selectivity towards cis-α-irone. 24 residues including the control G85 (highlighted in red outline) were subjected to site-saturation mutagenesis, using pMT1 as the template. 24 mutant libraries and 1 non-mutated wild-type (WT, highlighted in green outline) pMT1 were transformed into *E. coli* for pooled-colony reaction. Trans-α-irone and cis-α-irone produced from each pooled mutant library were quantified. Fold change was calculated by dividing the cis-α-irone produced from one reaction with cis-α-irone produced by G85m reaction. Similarly, cis-α-irone to trans-α-

irone ratio (cis-to-trans-α-irone ratio) was also quantified and fold change was calculated against G85m control. **c**, Pooled-colony reaction to identify the residue(s) to mutate to improve pMT6 selectivity towards cis-α-irone. 18 residues including the control E153 (highlighted in red outline) were subjected to site saturation mutagenesis using pMT6 as template. 18 mutant libraries and pMT6 (highlighted in green outline) were transformed into *E. coli* for pooled-colony reaction. Cis-α-irone and β-irone were quantified. Fold change was calculated by dividing the cis-α-irone or β-irone produced from one reaction with cis-α-irone or β-irone produced by E153m reaction, respectively. Similarly, β-irone to cis-α-irone ratio (β-to-cis-a-irone ratio) was also quantified.

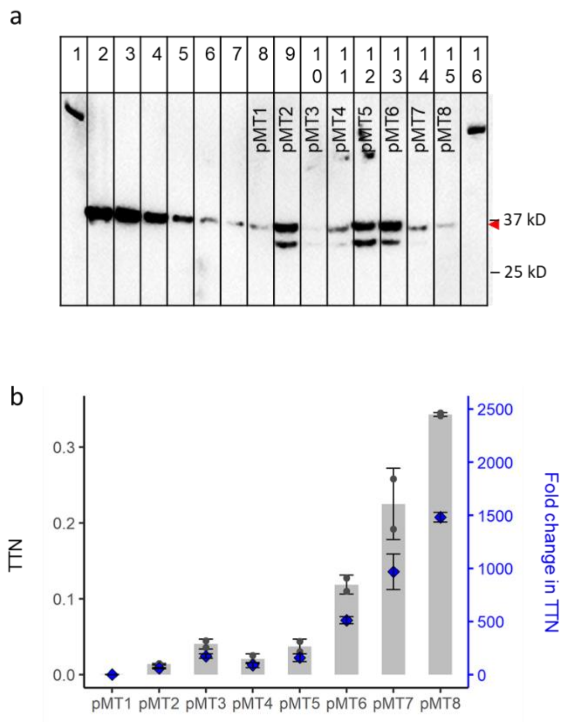


**Supplementary Figure 3**. Structure-guided engineering of pMT1 to produce cis-α-irone.

**a**, Western blot analysis of the soluble amount of pMT mutant enzymes taken from equal volume of cell lysate. Lanes 1 and 16 are the molecular marker with molecular weight indicated at the side. Lane 2–7 are purified known amount of pMT1 (2-fold dilution from 90 ng/μl to 2.8 ng/μl). The signal corresponding to pMT enzymes is indicated by the red arrow. The uncropped gel image data is provided as Supplementary Fig.14. **b**, The mean total turnover number (TTN) of each pMT mutant. TTN was obtained by dividing the concentration of cis-α-irone produced by the concentration of purified pMT enzyme. Fold change in TTN of all the mutants was measured against the TTN of pMT1, and is represented by blue diamonds. The average and the s.d. of two biologically independent experiments are shown.

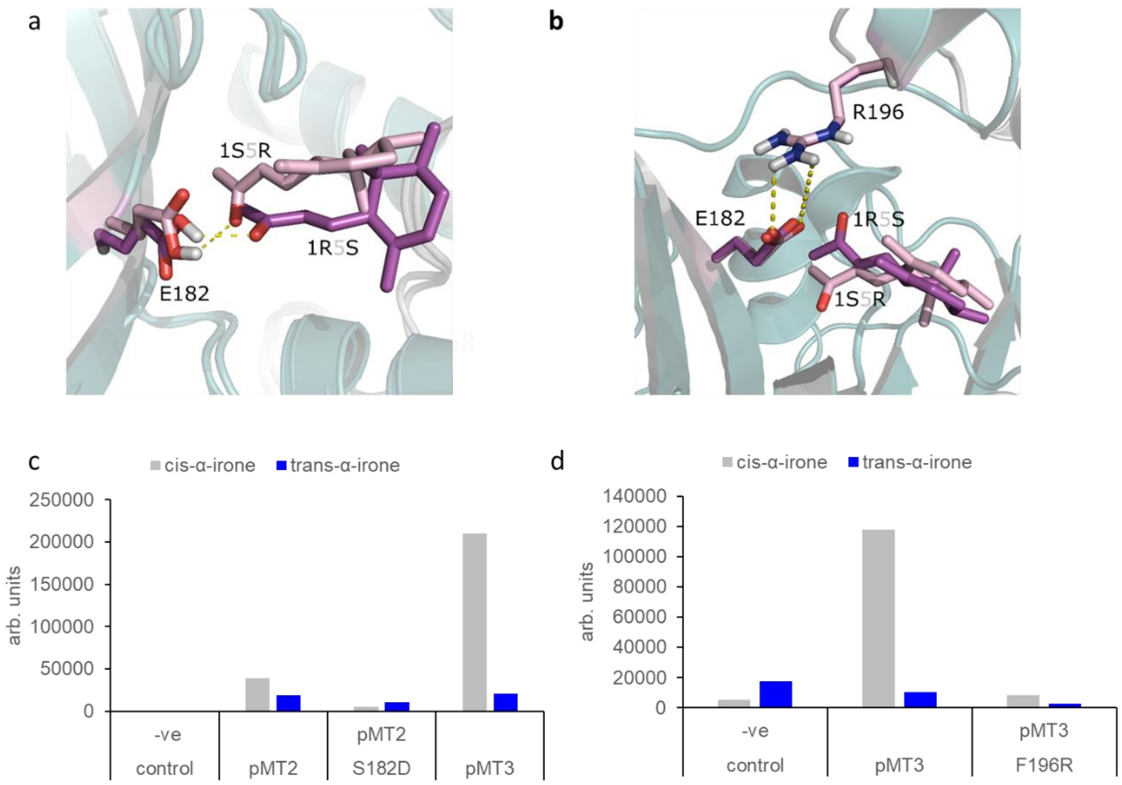


**Supplementary Figure 4**. Structural visualization of the mutant S182E (pMT3) and mutation study to explore the effect of S182E mutation.

**a**, Hydrogen bonds (yellow dashes) were formed between the protonated E182 residue and the ketone moiety of cis-α-irone (1R5S and 1S5R). Cis-α-irone was manually docked into the active site of pMT3 with energy minimization. The snapshot corresponds to the beginning of molecular dynamics (MD) simulation **b**, When F196 was mutated to R196, it competed with irone for hydrogen bonding with E182; thus, the activity of the enzyme was negatively affected. **c**, Bar chart representing the level of trans- and cis-α-irone detected in the headspace of the reaction catalyzed by negative (-ve) control (IspD), pMT2 (Y200F), pMT2-S182D (Y200F_S182D), and pMT3 (Y200F_S182E). When S182 was mutated to aspartic acid, hardly any cis-α-irone was detected; when S182 was mutated to glutamic acid, an increase in cis-α-irone was detected. **d**, Bar chart representing the level of trans- and cis-α-irone detected in the headspace of the reaction catalyzed by -ve control (IspD), pMT3 (Y200F_S182E) and pMT3-F196R (Y200F_S182D_F196R). Cis-α-irone level was drastically decreased when F196 was mutated to arginine on top of pMT3. arb. units: arbitrary units.

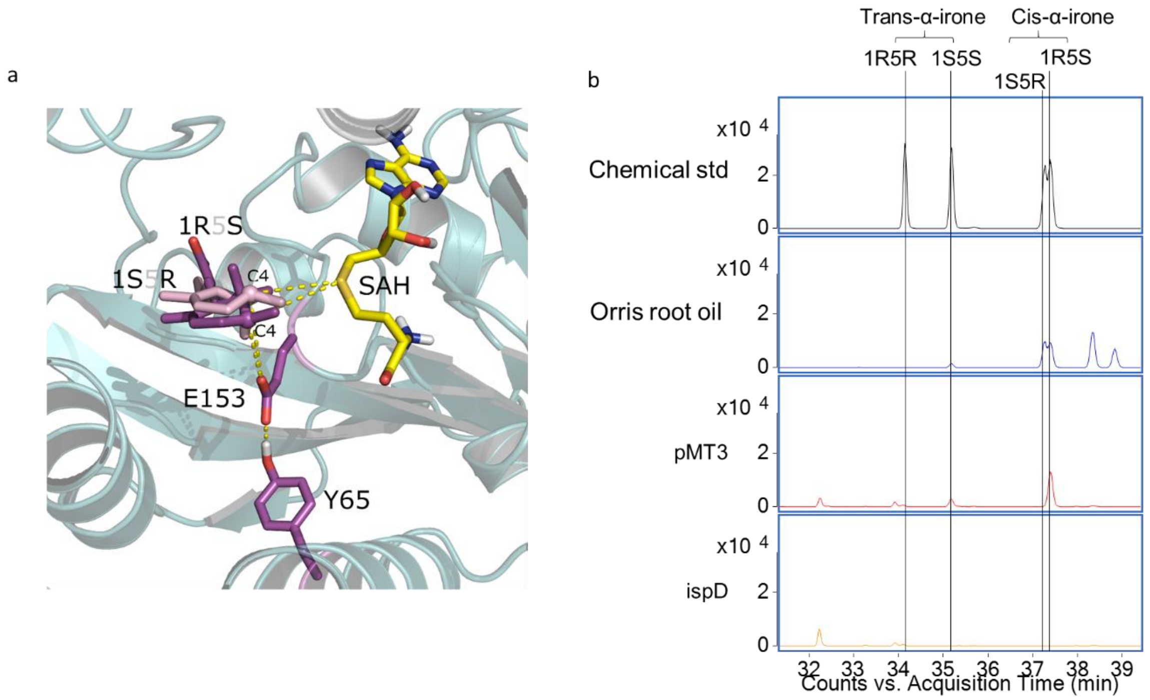


**Supplementary Figure 5.** Structural visualization of active residue E153 based on pMT3.

**a**, pMT3 model is shown as a cartoon representation. The residues E153, Y65, irone isomers are displayed as magenta sticks and SAH cofactor is displayed as yellow sticks. The interaction network for the reactivity is highlighted by yellow dashes, where the distances are on average 4-5 Å between the C4 carbon and the Sulphur of SAH or the oxygen of E153. The hydrogen bond between Y65 and E153 is around 2.0Å. The snapshot was taken from the beginning of the MD simulation. **b**, chiral GC analysis of the α-irone isomers from synthetic standard, orris root oil, reactions by pMT3 and IspD (negative control). The peaks corresponding to the four α-irone isomers were labelled. Clearly, all four isomers are present in chemical standard. Three isomers were detected in orris root oil with both cis-α-irone (1S5R, 1R5S) being the major irone constituents. Only two irone isomers were detected in the reaction by pMT3, with cis-α-irone (1R5S, the finest iris-like note) being the predominant product.

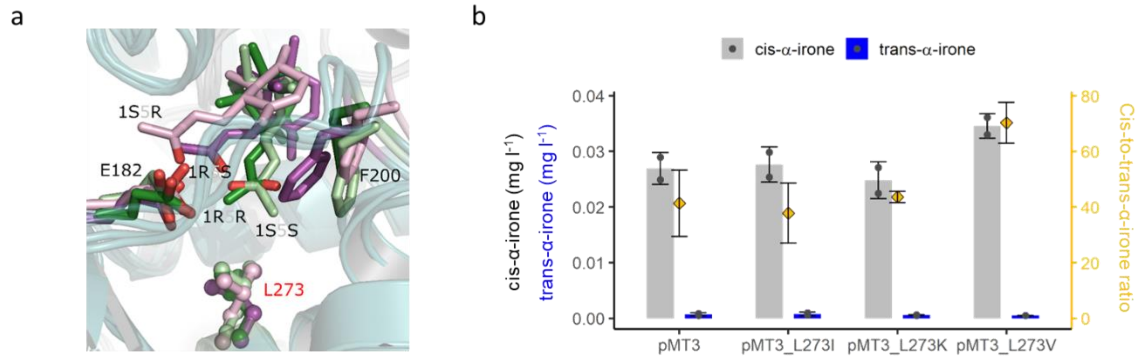


**Supplementary Figure 6.** Structural visualization of the interaction between L273 and irone and mutagenesis to identify the best mutant at L273.

**a**, Interactions between four isomers of irone and L273. cis isomers (1R5S and 1S5R) are shown in magenta whereas trans isomers (1R5R and 1S5S) are shown in green. E182, F200 and L273 form a structural environment to interact mostly with trans-α-irones. **b**, Bar chart representing the average concentration of trans- and cis-α-irone detected in the reaction catalysed by pMT3 (Y200F_S182E), pMT3_L273I, pMT3_L273K and pMT3_L273V. Cis-to-trans-α-irone ratio is shown as orange diamonds. The average and the s.d. of two biologically independent experiments are shown. L273V mutation gave rise to the highest cis-α-irone and the lowest trans-α-irone production.

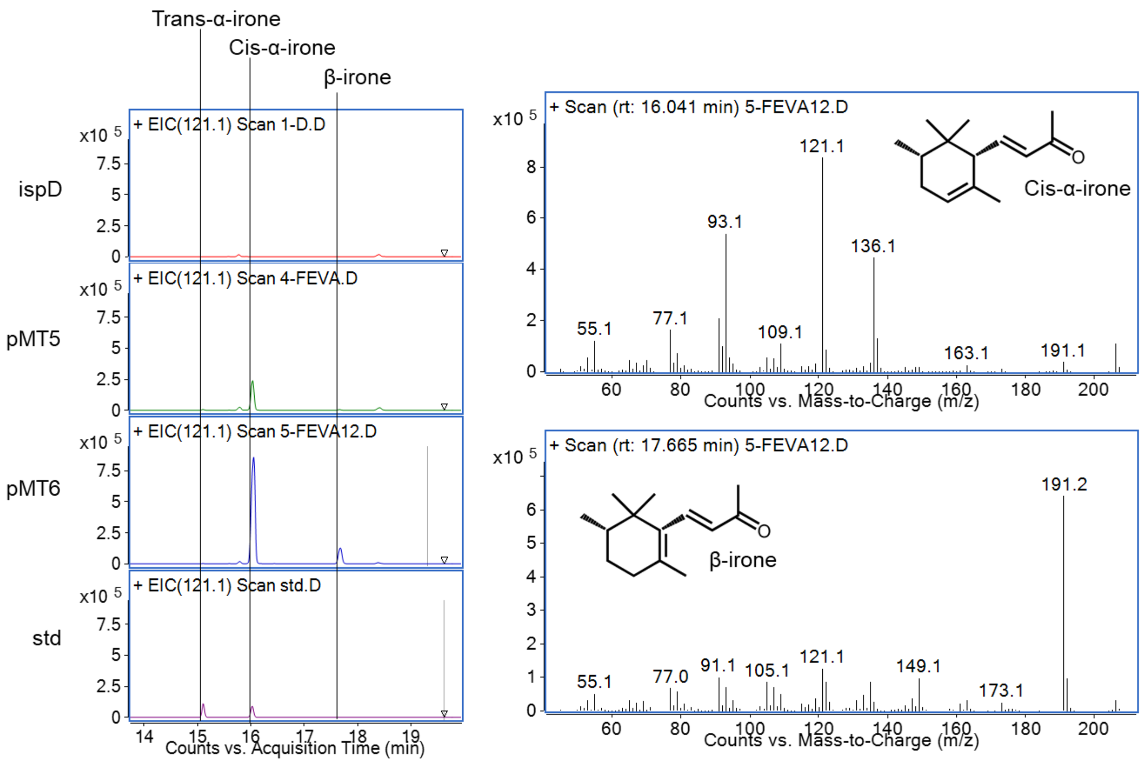


**Supplementary Figure 7.** HS-SPME-GCMS analysis to identify the products in pMT5 and pMT6 reaction.

Cell lysates overexpressing IspD (negative control) pMT5, pMT6 were used to assay against psi-ionone. The reaction mixtures were subjected to HS-SPME-GCMS analysis. An additional methylated product corresponded to β-irone was detected in the reaction containing pMT6. The mass spectrum of cis-α-irone and β-irone are shown. The retention time and mass spectrum of the irones detected are the same as the synthetic chemical standard (std) from Sigma Aldrich.

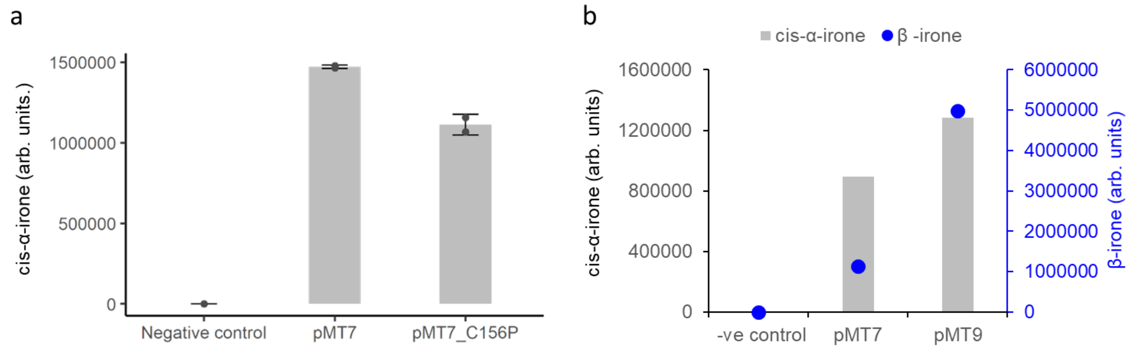


**Supplementary Figure 8.** Mutagenesis study based on pMT7.

**a**, Level of cis-α-irone detected in the headspace of reactions catalyzed by negative (-ve) control (IspD), pMT7, and pMT7_C156P. The average and the s.d. of two biologically independent experiments are shown. **b**, Level of cis-α-irone and β-irone detected in the headspace of reactions catalyzed by -ve control (IspD), pMT7 and pMT9 (pMT7_C156A). arb. units: arbitrary units.

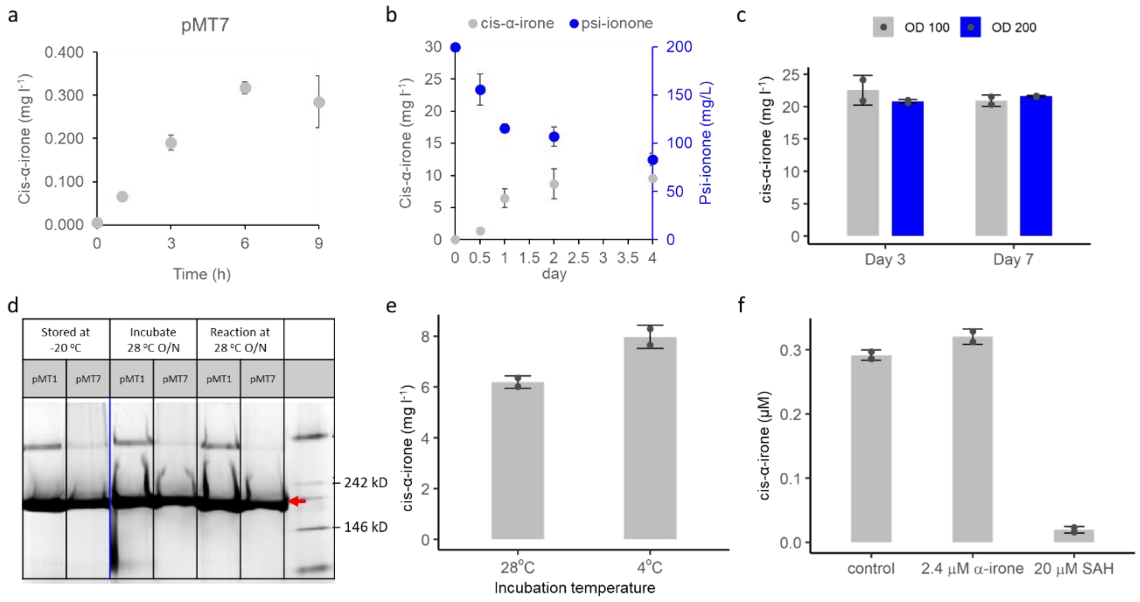


**Supplementary Figure 9.** Activity and stability analysis of pMT7.

**a**, Time course of cis-α-irone production by purified pMT7. The average and the s.d. of three biologically independent experiments are shown. **b**, Time course of cis-α-irone production and psi-ionone consumption by pMT7 in cell extracts. **c**, The final amount of cis-α-irone produced by pMT7 in cell extracts when amount of cell extracts or duration of incubation were varied. **d**, Native PAGE gel analysis of oligomeric state of pMT1 or pMT7 enzyme when stored at -20 $^{o}$C, incubated at 28 $^{o}$C overnight (O/N), or reacted at 28 $^{o}$C overnight (O/N). The blue line delineates the boundary of two non-adjacent lanes. The last lane is molecular marker with size indicated at the side. The uncropped gel image is provided as Supplementary Fig.15. The experiment was repeated once with similar results obtained. The expected protein band is indicated by the red arrow. **e**, Final amount of cis- α-irone produced by pMT7 in cell extracts by overnight pre-incubating at 28 $^{o}$C or 4 $^{o}$C. **f**, Cis-α-irone produced by purified pMT7 when 20 µM SAH or 2.4 µM irone was added into the reaction. For **b**, **c**, **e,** and **f**, The average and the s.d. of two biologically independent experiments are shown.

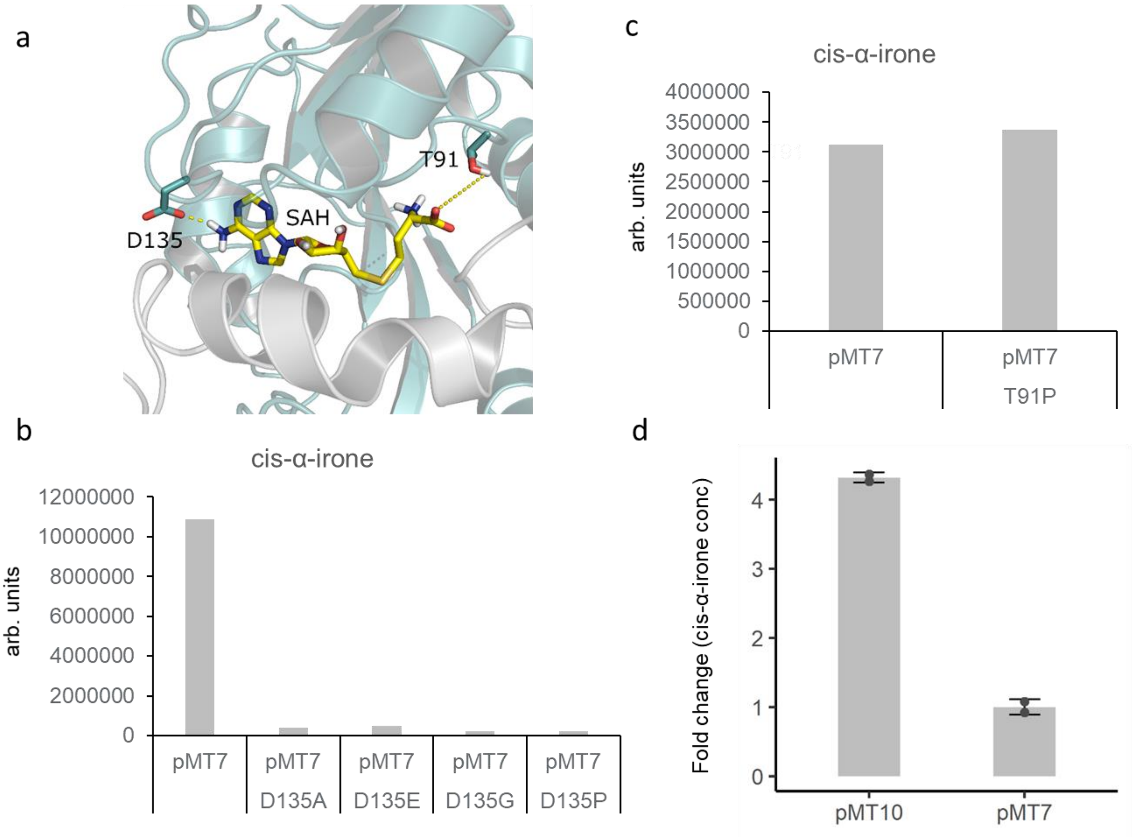


**Supplementary Figure 10.** Further mutations to improve pMT7 activity and reduce SAH inhibition.

**a**, Structural visualization of the hydrogen bond network (yellow dashes) between SAH and residue D135 and T91 from pMT3 model. A stronger hydrogen bond network is expected between SAH and D135. **b**, Level of cis-α-irone detected in the headspace of reactions catalyzed by pMT7, pMT7_D135A, pMT7_D135E, pMT7_D135G, and pMT7_D135P. Cis-α-irone levels were drastically reduced when D135 was mutated. **c**, Level of cis-α-irone detected in the headspace of reactions catalyzed by pMT7 and pMT7_T91P. Cis-α-irone level was slightly enhanced when T91 was mutated to Proline. T91P mutation was suggested by Hotspot Wizard 3[2]. **d,** Bar chart represents the average fold change in cis-α-irone concentration produced by cell lysate overexpressing pMT10 as compared to cell lysate overexpressing pMT7. The average and the s.d. of two biologically independent experiments are shown. arb. units: arbitrary units.

a

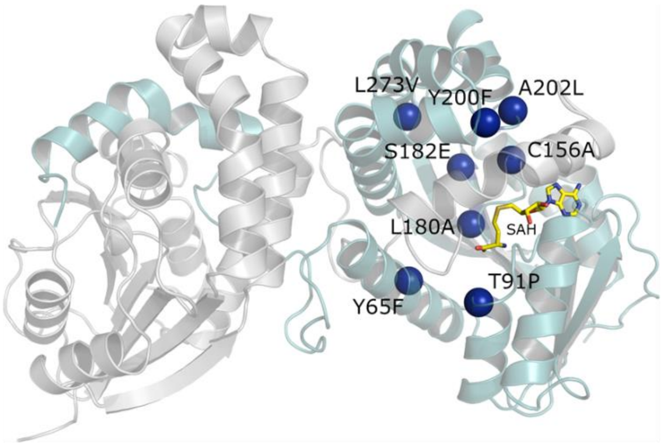


b

| Name | Mutations | | | | | | | |
|---|---|---|---|---|---|---|---|---|
| pMT2 | Y200F | | | | | | | |
| pMT3 | Y200F | S182E | | | | | | |
| pMT4 | Y200F | S182E | L273V | | | | | |
| pMT5 | Y200F | S182E | L273V | L180A | | | | |
| pMT6 | Y200F | S182E | L273V | L180A | A202L | | | |
| pMT7 | Y200F | S182E | L273V | L180A | A202L | Y65F | | |
| pMT8 | Y200F | S182E | L273V | L180A | A202L | | C156P | |
| pMT9 | Y200F | S182E | L273V | L180A | A202L | Y65F | C156A | |
| pMT10 | Y200F | S182E | L273V | L180A | A202L | Y65F | C156A | T91P |

**Supplementary Figure 11**. Summary information of the mutations from pMT1 to pMT10.

**a**, Structural visualization of residues mutated from pMT1 to pM10. The mutated residues are labelled as blue spheres and the mutations are labelled. The cofactor SAH is shown as yellow sticks. **b**, A summary of the mutations from pMT2 to pMT10. The sequences are provided as Supplementary data 3.

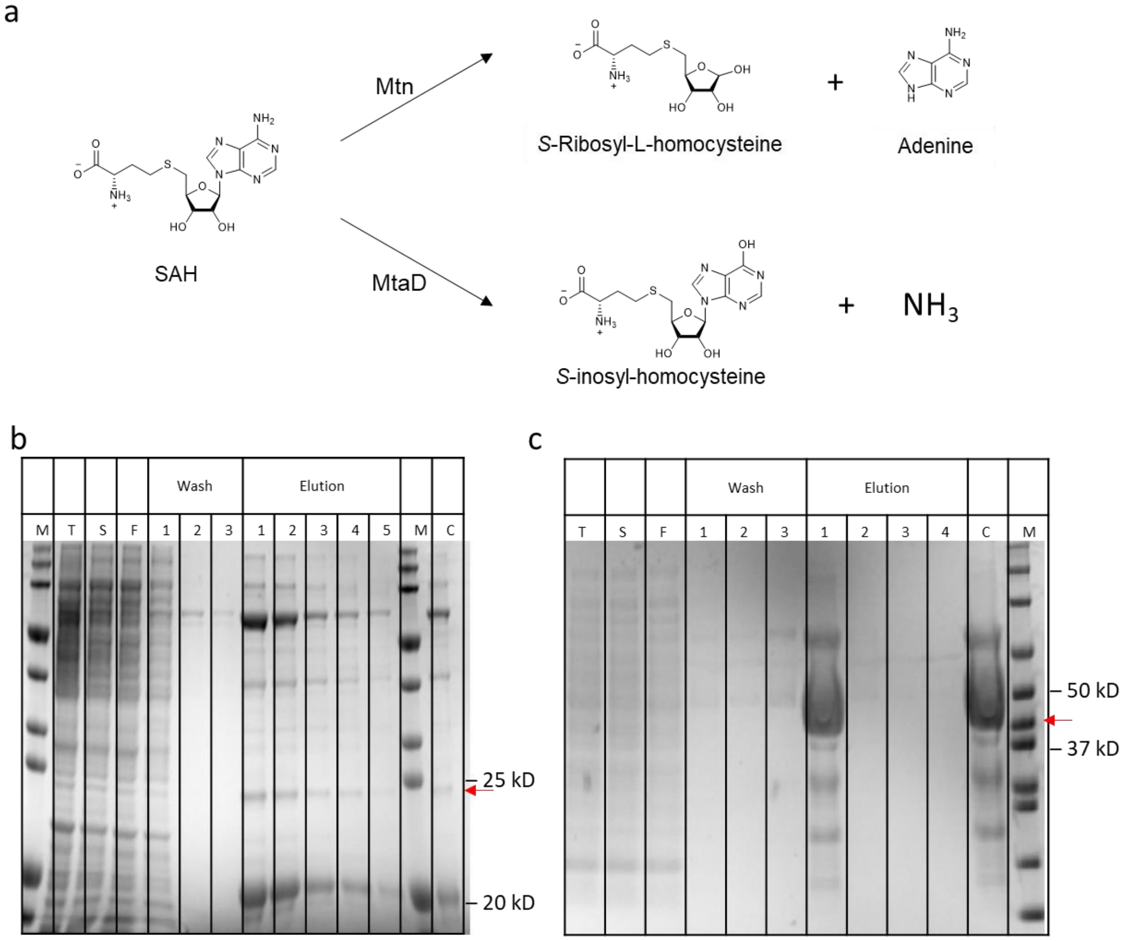


**Supplementary Figure 12.** Auxiliary reactions to remove SAH inhibition to pMT reactions.

**a**, Schematic representation of reactions catalyzed by Mtn and MtaD. Mtn hydrolyses SAH into *S*-ribosyl-L-homocysteine and adenine. MtaD deaminates SAH to *S*-inosyl-homocysteine and ammonia. SDS-PAGE gel analysis of purification of **b**, Mtn and **c**, MtaD. The protein is indicated by the red arrow. The lane labels are as follows. M: marker with molecular weight indicated at the side. T: total protein. S: soluble protein. F: flowthrough after his-tag binding. C: concentrated protein by combining all the elution fraction and desalting by ultracentrifugation. The raw data for the gel images are provided as Supplementary Fig. 16 and 17.

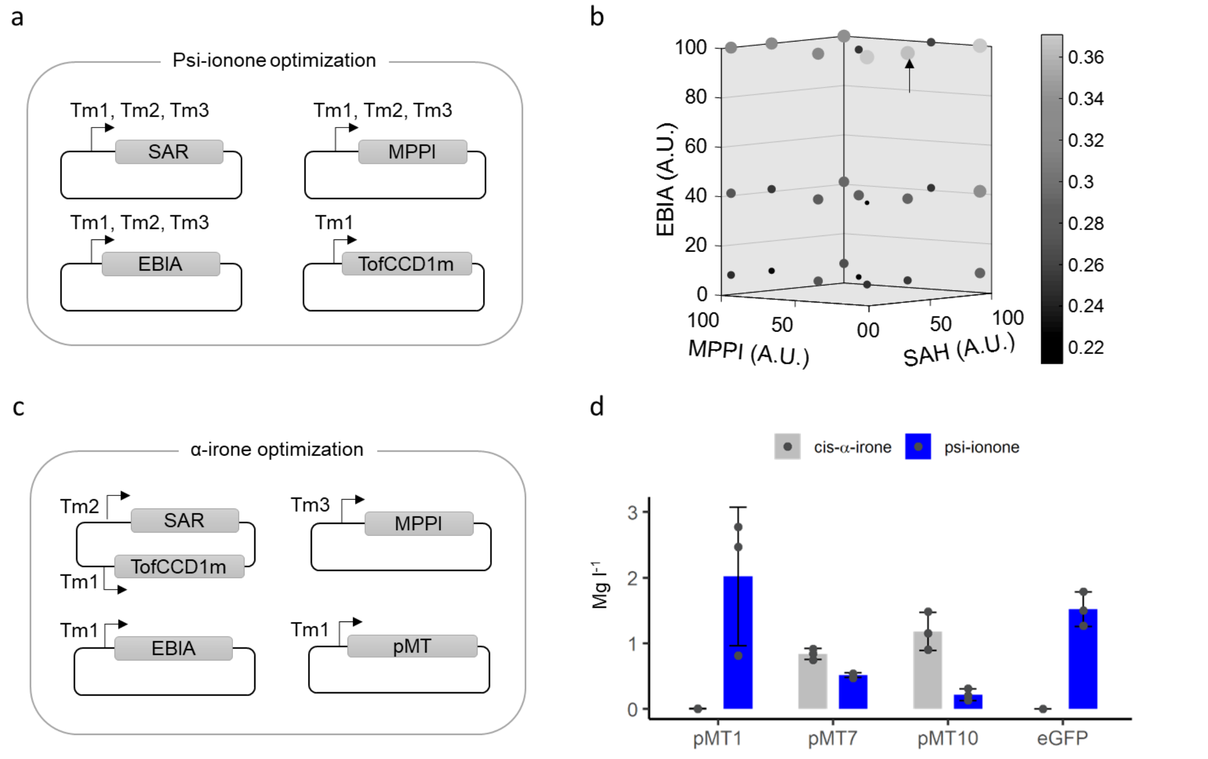


**Supplementary Figure 13.** Optimizing *in vivo* production of psi-ionone and cis- α-irone from simple carbon source.

**a**, Schematic representation of the plasmids used to optimize psi-ionone production. Four plasmids were used. The first plasmid carried the SAR module, which contained the upper mevalonate enzymes: HMG-CoA synthase (HmgS), Acetoacetyl-CoA thiolase (AtoB), and truncated HMG-CoA reductase (tHmgR). The second plasmid carried the MPPI module, which contained the lower mevalonate enzymes: mevalonate kinase (MevK), phosphomevalonate kinase (PMK), mevalonate pyrophosphate decarboxylase (PMD), and IPP isomerase (Idi). The third plasmid carried the lycopene synthesis EBIA module: GGPP synthase (CrtE), phytoene synthase (CrtB), phytoene desaturase (CrtI), and FPP synthase (IspA). The last plasmid carried the modified OfCCD1 enzyme fused with thioredoxin (TofCCD1m)[3]. Tm1, Tm2, and Tm3 are mutated T7 promoters with different strengths (Tm1 > Tm2 > Tm3)[4]. **b**, Multidimensional heuristics process (MHP) was used to optimize psi-ionone specific titer (mg $l^{-1}$ $OD^{-1}$) by tuning the promoters controlling the SAR, MPPI and EBIA modules while keeping the promoter for TofCCD1m as Tm1[4]. The highest specific psi-ionone titer obtained were from strain 2311 (Tm2-SAH, Tm3-MPPI, Tm1-EBIA, Tm1-TofCCD1m) as indicated by the black arrow. To reduce the number of plasmids, the 1$^{st}$ and 4$^{th}$ module were combined into one plasmid and the strain was further named to 2O31. **c**, Schematic representation of the plasmids used to optimize cis- α-irone production *in vivo*. Four plasmids were used. The first plasmid contained both the SAR module and TofCCD1m. The second plasmid carried MPPI module. The third plasmid carried EBIA module. The fourth plasmid carried the pMT enzyme. **d**, Bar chart represents the average amount of cis- α-irone and psi-ionone produced when pMT1, pMT7, pMT10, or eGFP was overexpressed on the fourth plasmid. The average and the s.d. of three biologically independent experiments are shown.

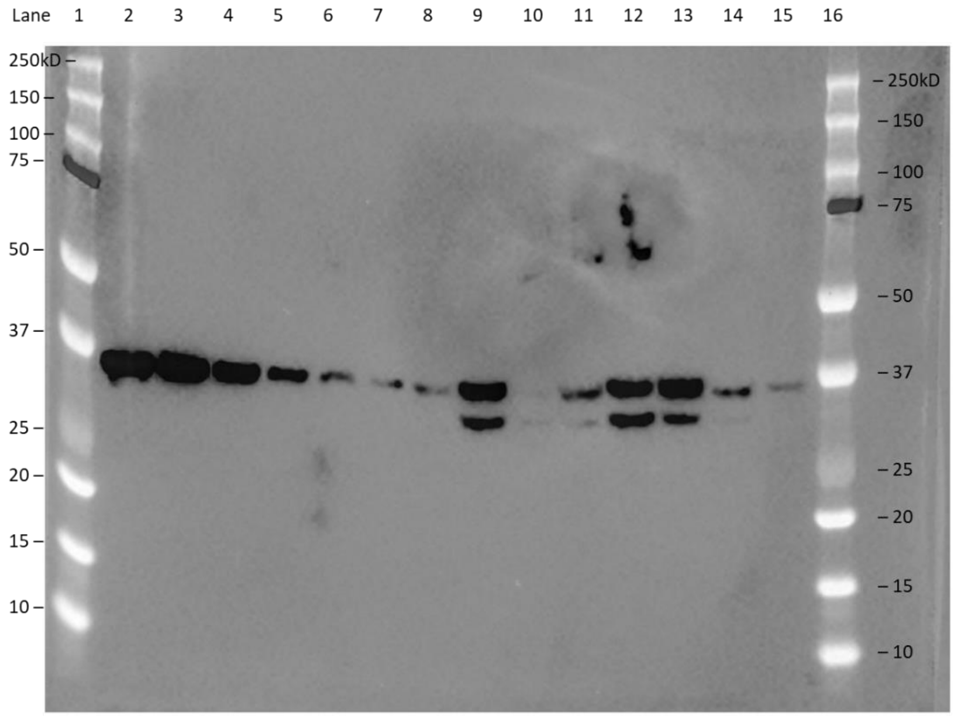


**Supplementary Figure 14.** The uncropped western blot image corresponding to Supplementary Fig. 3a.

The gel image is an overlay of two images: one was taken with white trans illumination and the other was taken with chemiluminescence with high sensitivity. The antibody used is HRP Mouse monoclonal Anti-6X His tag® antibody [GT359] (ab184607). The antibody is conjugated with HRP and is species independent. The antibody is diluted 2000 times in tris-buffered saline with 0.1% Tween® 20 detergent (TBST) buffer with 1% milk. The legend is as follows. Lanes 1 and 16 are Precision Plus Protein™ Dual Color Standards (Bio-Rad) with labelled molecular weight. 4 µl of the ladder was loaded. Lanes 2–7 are standard curves of purified his-tagged pMT enzymes at 10 µl each with the following pre-determined concentrations. Lane 2: 90 ng $\mu l^{-1}$; Lane 3: 45 ng $\mu l^{-1}$; Lane 4: 22.5 ng $\mu l^{-1}$; Lane 5: 11.25 ng $\mu l^{-1}$; Lane 6: 5.625 ng $\mu l^{-1}$; Lane 7: 2.8125 ng $\mu l^{-1}$. Lanes 8–15 are pMT mutant enzymes in cell lysate at 10 µl each. Lane 8: pMT1; Lane 9: pMT2; Lane 10: pMT3; Lane 11: pMT4; Lane 12: pMT5; Lane 13: pMT6; Lane 14: pMT7; Lane 15: pMT8.

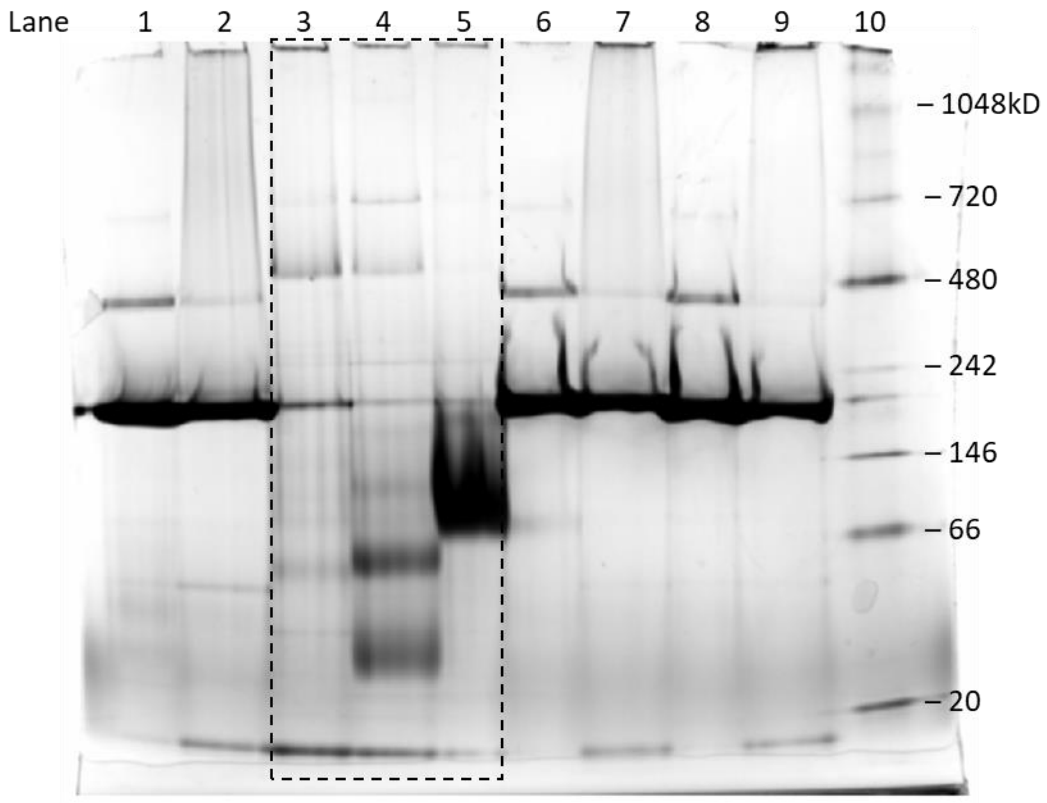


**Supplementary Figure 15.** The uncropped native polyacrylamide gel electrophoresis (PAGE) gel image corresponding to Supplementary Fig. 9d.

Lane 1: pMT1 enzyme stored at -20 $^oC$. Lane 2: pMT7 enzyme stored at -20 $^oC$. Lanes 3 to 5 have been removed from Supplementary Fig. 9d, which is boxed in dotted line. Lane 6: pMT1 incubated overnight at 28 $^oC$. Lane 7: pMT7 incubated overnight at 28 $^oC$. Lane 8: pMT1 reacted overnight at 28 $^oC$. Lane 9: pMT7 reacted overnight at 28 $^oC$. Lane 10: NativeMark™ Unstained Protein Standard (ThermoFisher Scientific) with labelled molecular weight.

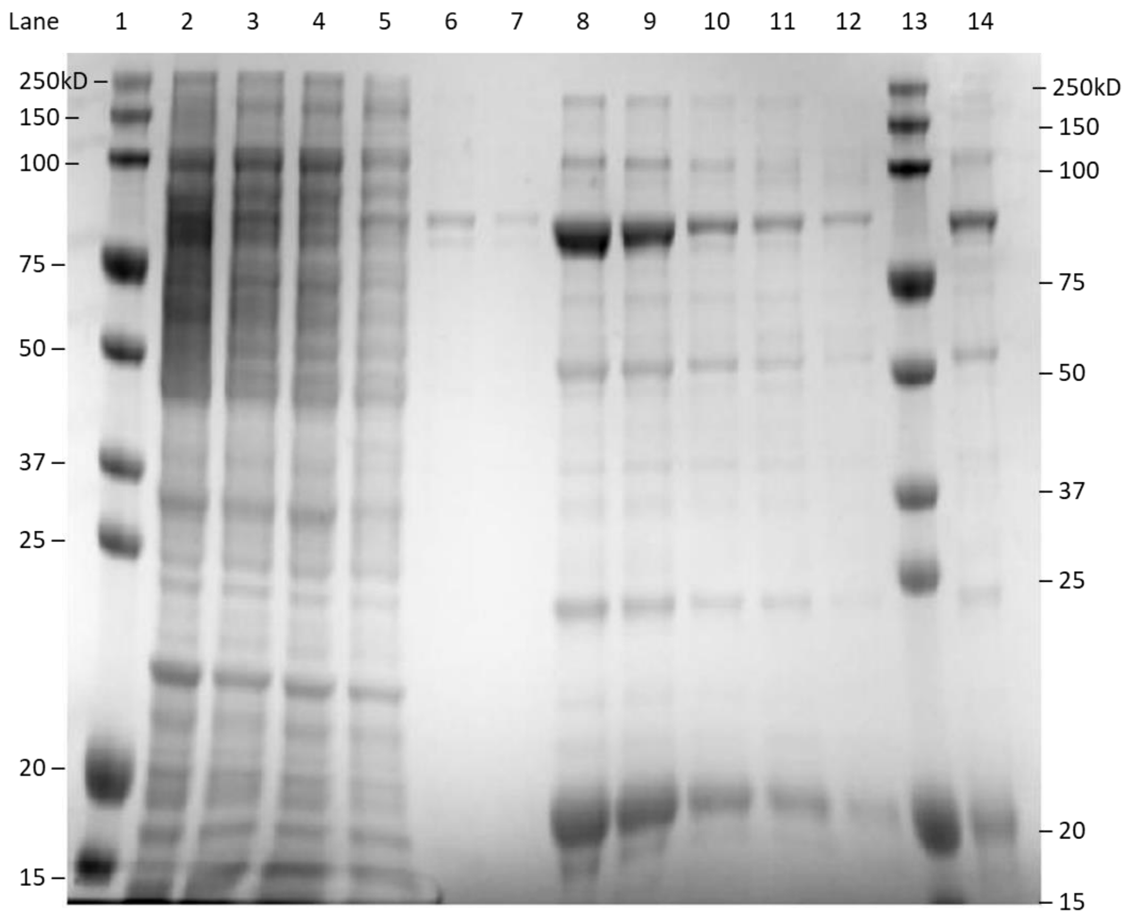


**Supplementary Figure 16.** The uncropped sodium dodecyl sulfate–polyacrylamide gel electrophoresis (SDS-PAGE) gel image corresponding to Supplementary Fig. 12b.

Lanes 1 and 13: Precision Plus Protein™ Dual Color Standards (Bio-Rad) with labelled molecular weights. Lane 2: total protein. Lane 3: soluble protein. Lane 4: flowthrough after his-tag binding. Lanes 5–7: flowthrough with wash buffer. Lanes 8–12: eluted protein. Lane 14: concentrated protein by combining all the elution fraction and desalting by ultracentrifugation.

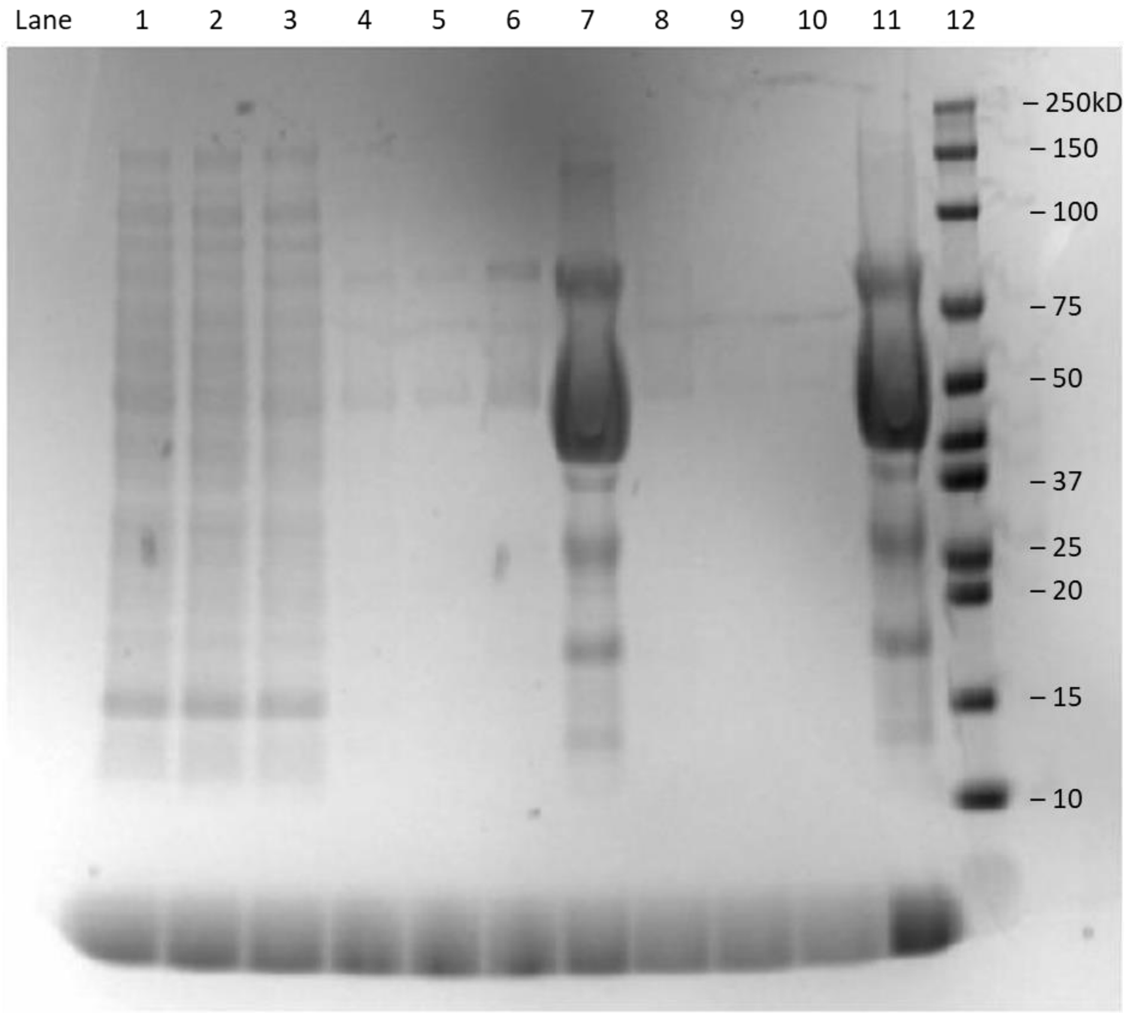


**Supplementary Figure 17.** The uncropped sodium dodecyl sulfate–polyacrylamide gel electrophoresis (SDS-PAGE) gel image corresponding to Supplementary Fig. 12c.

Lane 1: total protein. Lane 2: soluble protein. Lane 3: flowthrough after his-tag binding. Lanes 4–6: flowthrough with wash buffer. Lanes 7–10: eluted protein. Lane 11: concentrated protein by combining all the elution fraction and desalting by ultracentrifugation. Lane 12: Precision Plus Protein™ Dual Color Standards (Bio-Rad) with labelled molecular weight.

## Supplementary tables

**Supplementary Table 1**. Selected methyltransferases with known structures were tested for their potential activity for conversion of psi-ionone to irone. TleD is the only enzyme among the 4 methyltransferases that fulfil all the selection criteria. Irone was detected in the headspace of the TleD reaction with psi-ionone. Ref: supplementary reference.

| Enzyme | Protein name | PDB ID | Methylation | Cyclization | Natural substrate contains terpene moiety? | Forms 6-member ring? | TTN | Ref |
|---|---|---|---|---|---|---|---|---|
| Cyclopropane mycolic acid synthase 1 | Mt.CMAS | 1KPG | Yes | Yes | No | No | ND | Ref [5] |
| Geranyl diphosphate 2-C-methyltransferase | Sl.GdpMT | 4F86 | Yes | No | Yes | No | ND | Ref [6] |
| Methyltransferase-like protein | Ss.SpnF | 4PNE | No | Yes | No | No | ND | Ref [7] |
| O-methltransferase | TleD (pMT1) | 5GM2 | Yes | Yes | Yes | Yes | <0.001 | Ref [8] |

**Supplementary Table 2.** Free Energy contributions per residue computed from MM/GBSA approaches[9]. Free energy calculations were performed using MD simulations from pMT1/3/4 in complex with each of the four isomers of α-irone. Energy profile change of the catalytic residue E153 is highlighted by coloring favorable contributions in blue and unfavorable in red. pMT1 corresponds to the wild-type whereas pMT3 is the Y200F/S182E mutant and pMT4 the Y200/S182E/L273V mutant. The numbering of residues corresponds to the X-ray structure (PDB id: 5GM2)[8]. Free energy values are given in kcal $mol^{-1}$ and correspond to the mean from 100 snapshots extracted uniformly. The initial and final structures for MM/GBSA analysis are provided as Supplementary data 2.

| | E153 | | | Y200 | | | S182 | | | L273 | | |
|---|---|---|---|---|---|---|---|---|---|---|---|---|
| | pMT1 | pMT3 | pMT4 | pMT1 | pMT3 | pMT4 | pMT1 | pMT3 | pMT4 | pMT1 | pMT3 | pMT4 |
| cis/1R5S | 1.14 | -0.36 | -0.1 | -1.82 | -2.14 | -1.63 | -0.19 | -0.88 | -0.22 | -0.09 | -0.05 | -0.53 |
| cis/1S5R | -0.09 | -0.18 | -0.6 | -1.29 | -0.90 | -0.95 | -0.79 | -1.01 | -0.39 | -0.29 | -0.23 | -0.14 |
| trans/1R5R | -0.20 | -0.16 | 0.11 | -1.45 | -1.44 | -1.45 | -0.38 | -0.90 | -1.37 | -0.23 | -0.10 | -0.07 |
| trans/1S5S | -0.03 | 0.00 | 0.70 | -2.07 | -2.14 | -2.12 | -0.30 | -1.40 | -1.88 | -0.09 | -0.33 | -0.34 |

**Supplementary Table 3.** Strains and plasmids used in the study. The heterologous sequence data are provided in Supplementary data 3.

| Name | Description | Reference | Remarks |
|---|---|---|---|
| *E. coli* BL21-Gold (DE3) | $F^{-}$ *ompT hsdS* ($r_B^-$ $m_B^-$) *dcm*$^{+}$ *Tet*$^{r}$ *gal* λ(DE3) *endA* Hte | Stratagene | For *in vitro* enzyme expression |
| *E. Coli* Stellar Competent Cells | F-, *endA1, supE44, thi-1, recA1, relA1, gyrA96, phoA,* Φ80d *lacZ*Δ M15, Δ(*lacZYA-argF*) U169, Δ(*mrr-hsdRMS-mcrBC*), Δ*mcrA*, λ- | Clontech | For plasmid construction |
| 2O31 strain | BL21, Δ*aroA*, Δ*aroB*, Δ*aroC*, Δ*serC*, carrying plasmids p15A-spec-Tm2-hmgS-atoB-hmgR-Tm1-OfCCD1m, p15A-cam-Tm3-mevK-pmk-pmd-idi, p15A-kan-Tm1-crtEBI-ispA. | This study | Auxotrophic strain. Base strain to produce psi-ionone and transform pMT enzymes. |
| 2O31 pMT10 | 2O31 strain transformed with p15A-amp-Tm1-pMT10 | This study | Strain for α-irone production. |
| 2O31 pMT10r | 2O31 strain transformed with p15A-amp-Tm1-pMT10-metK-mtn | This study | Strain for α-irone production. |
| 2O31 ΔmetJ pMT10 | 2O31 strain with *metJ* deletion and transformed with p15A-amp-Tm1-pMT10 | This study | Strain for α-irone production. |
| 2O31 ΔmetJ pMT10r | 2O31 strain with *metJ* deletion and transformed with p15A-amp-Tm1-pMT10-metK-mtn | This study | Strain for α-irone production. |
| p15A-spec-Tm2-hmgS-atoB-hmgR-Tm1-OfCCD1m | Plasmid for overexpression of *hmgs, atoB, thmgR* genes controlled by a mutated Tm2 promoter, and modified *OfCCD1,* controlled by a mutated Tm1 promoter. It carries the spectinomycin resistance gene. | Ref[3] | Module 1 and module 4 |

| | | | |
|---|---|---|---|
| p15A-cam-Tm3-mevK-pmk-pmd-idi | Plasmid for overexpression of *mevK*, *pmk, pmd, idi* genes, controlled by a Tm3 promoter. It carries the chloramphenicol resistance gene. | Ref[3] | Module 2 |
| p15A-kan-Tm1-crtEBI-ispA | Plasmid for overexpression of *crtE, crtB, crtI* and *ispA* genes, controlled by a Tm1 promoter. It carries the kanamycin resistance gene. | Ref[3] | Module 3 |
| p15A-amp-Tm1-eGFP | Plasmid for overexpression of the *eGFP* gene, controlled by a Tm1 promoter. It carries the ampicillin resistance gene. | This study | Module 5 |
| p15A-amp-Tm1-pMT1 | Plasmid for overexpression of *pMT1* gene, controlled by a Tm1 promoter. It carries the ampicillin resistance gene. | This study | Module 5 |
| p15A-amp-Tm1-pMT7 | Plasmid for overexpression of *pMT7* gene, controlled by a Tm1 promoter. It carries the ampicillin resistance gene. | This study | Module 5 |
| p15A-amp-Tm1-pMT10 | Plasmid for overexpression of *pMT10* gene, controlled by a Tm1 promoter. It carries the ampicillin resistance gene. | This study | Module 5 |
| p15A-amp-Tm1-pMT10-metK-mtn | Plasmid for overexpression of *pMT10, metK, mtn* genes, controlled by a Tm1 promoter. It carries the ampicillin resistance gene. | This study | Module 5 |

**Supplementary Table 4.** Summary of structure-guided evolution of pMT enzyme to produce cis-α-irone from psi-ionone.

| Round | Name | Parent | Diversification strategy | Changes made |
|---|---|---|---|---|
| 1 | pMT2 | pMT1 or TleD from *Streptomyces blastmyceticus* | Pooled colony screening to increase cis-α-irone production. Site-saturation mutagenesis, Y200X | Y200F |
| 2 | pMT3 | pMT2 | Pooled colony screening to increase cis/trans-α-irone ratio. Site-saturation mutagenesis, S182X | S182E |
| 3 | pMT4 | pMT3 | Computer-aided structural analysis and site-saturation mutagenesis, L273X | L273V |
| 4 | pMT5 | pMT4 | Pooled colony screening to increase cis-α-irone production. Site-saturation mutagenesis, L180X | L180A |
| 5 | pMT6 | pMT5 | Structural analysis to reduce the binding pocket,<br>A202S, A202V, A202L, A202F | A202L |
| 6 | pMT7 | pMT6 | Pooled colony screening to reduce β-irone production. Site-saturation mutagenesis,<br>Y65X | Y65F |
| 7 | pMT8 | pMT6 | Pooled colony screening to reduce β-irone production. Site-saturation mutagenesis,<br>C156X | C156P |
| 8 | pMT9 | pMT7 | Combined positive C156X mutation on top of pMT7,<br>C156A, C156P | C156A |
| 9 | pMT10 | pMT9 | Removed the hydrogen bond between SAH and pMT enzyme. Stability analysis by Hotspot Wizard 3.0[2]. T91P | T91P |

**Supplementary Table 5.** Comparison of yield by natural extraction and biomanufacturing methods to produce cis-α-irone. Biomanufacturing method is 3,800-18,000-fold more efficient as compared to natural extraction given the same land area and duration.

| Manufacturing methods | Extraction (Native pathway) | Biomanufacturing (This work) |
|---|---|---|
| Natural? | Yes | Yes |
| Stereoisomers | 3 | 2 |
| Production period | 3-6 years | 7-day fermentation<br>1 year for glucose production |
| Yield | 30-70 mg $kg^{-1}$ Orris root | 360 mg $kg^{-1}$ glucose |
| Land area / kg raw material | 10 $m^2$ | 0.04 $m^2$ |

## Supplementary References


1. Studier, F. W. Protein production by auto-induction in high-density shaking cultures. *Protein Expression and Purification* **41**, 207–234 (2005).
2. Sumbalova, L., Stourac, J., Martinek, T., Bednar, D. & Damborsky, J. HotSpot Wizard 3.0: web server for automated design of mutations and smart libraries based on sequence input information. *Nucleic Acids Res* **46**, W356–W362 (2018).
3. Chen, X., Shukal, S. & Zhang, C. Integrating Enzyme and Metabolic Engineering Tools for Enhanced α-Ionone Production. *J. Agric. Food Chem.* **67**, 13451–13459 (2019).
4. Zhang, C., Seow, V. Y., Chen, X. & Too, H.-P. Multidimensional heuristic process for high-yield production of astaxanthin and fragrance molecules in Escherichia coli. *Nat Commun* **9**, 1–12 (2018).
5. Huang, C., Smith, C. V., Glickman, M. S., Jacobs, W. R. & Sacchettini, J. C. Crystal Structures of Mycolic Acid Cyclopropane Synthases from Mycobacterium tuberculosis *. *Journal of Biological Chemistry* **277**, 11559–11569 (2002).
6. Ariyawutthiphan, O. *et al.* Structure analysis of geranyl pyrophosphate methyltransferase and the proposed reaction mechanism of SAM-dependent C-methylation. *Acta Cryst D* **68**, 1558–1569 (2012).
7. Fage, C. D. *et al.* The structure of SpnF, a standalone enzyme that catalyzes [4 + 2] cycloaddition. *Nature Chemical Biology* **11**, 256–258 (2015).
8. Yu, F. *et al.* Crystal structure and enantioselectivity of terpene cyclization in SAM-dependent methyltransferase TleD. *Biochem. J.* **473**, 4385–4397 (2016).
9. Miller, B. R. I. *et al.* MMPBSA.py: An Efficient Program for End-State Free Energy Calculations. *J. Chem. Theory Comput.* **8**, 3314–3321 (2012).
