# Supplementary material for: Total enzymatic synthesis of cis-α-irone from a simple carbon source
Source: Nat Commun. 2022 Dec 2;13:7421. doi: 10.1038/s41467-022-35232-2 (PMC9715568; doi:10.1038/s41467-022-35232-2)
Supplement: Supplementary file 3 — Description of Additional Supplementary Files [file 41467_2022_35232_MOESM3_ESM.pdf]

Description of Additional Supplementary Files

File name: Supplementary Data 1
Description: pdb files of the protein structures used for visualization and analysis for Fig. 2c-2e. Supplementary Fig. 4a, Fig. 4b, Fig. 5, Fig. 6a, Fig. 10 and Fig. 11a.

File name: Supplementary Data 2
Description: pdb files of the protein structures used for MM/GBSA computation for Supplementary Table 2.

File name: Supplementary Data 3
Description: An excel file listing heterologous gene sequences primers and gRNA used in the study.
This file contains:
Heterologous gene sequences used in this study.
Primers used in this work.
gRNA sequence for CRISPR-cas9 guided knockout
