# Supplementary material for: Total enzymatic synthesis of cis-α-irone from a simple carbon source
Source: Nat Commun. 2022 Dec 2;13:7421. doi: 10.1038/s41467-022-35232-2 (PMC9715568; doi:10.1038/s41467-022-35232-2)
Supplement: Supplementary file 7 — Reporting Summary [file 41467_2022_35232_MOESM7_ESM.pdf]

nature portfolio

Corresponding author(s): Xixian Chen
Isabelle André

Last updated by author(s): Nov 11, 2022

Reporting Summary

Nature Portfolio wishes to improve the reproducibility of the work that we publish. This form provides structure for consistency and transparency in reporting. For further information on Nature Portfolio policies, see our Editorial Policies and the Editorial Policy Checklist.

Statistics

For all statistical analyses, confirm that the following items are present in the figure legend, table legend, main text, or Methods section.

n/a	Confirmed	
☐	☒	The exact sample size (*n*) for each experimental group/condition, given as a discrete number and unit of measurement
☐	☒	A statement on whether measurements were taken from distinct samples or whether the same sample was measured repeatedly
☐	☒	The statistical test(s) used AND whether they are one- or two-sided<br>*Only common tests should be described solely by name; describe more complex techniques in the Methods section.*
☒	☐	A description of all covariates tested
☒	☐	A description of any assumptions or corrections, such as tests of normality and adjustment for multiple comparisons
☐	☒	A full description of the statistical parameters including central tendency (e.g. means) or other basic estimates (e.g. regression coefficient) AND variation (e.g. standard deviation) or associated estimates of uncertainty (e.g. confidence intervals)
☐	☒	For null hypothesis testing, the test statistic (e.g. *F*, *t*, *r*) with confidence intervals, effect sizes, degrees of freedom and *P* value noted<br>*Give P values as exact values whenever suitable.*
☒	☐	For Bayesian analysis, information on the choice of priors and Markov chain Monte Carlo settings
☒	☐	For hierarchical and complex designs, identification of the appropriate level for tests and full reporting of outcomes
☒	☐	Estimates of effect sizes (e.g. Cohen's *d*, Pearson's *r*), indicating how they were calculated

Our web collection on statistics for biologists contains articles on many of the points above.

Software and code

Policy information about availability of computer code

Data collection	Masshunter 10.0 (Agilent) was used to collect GCMS data. Lucullus 3.8 (Applikon) was used to collect bioreactor data. Gen5 (BioTek Synergy HT microplate reader) was used to collect biomass data. Image lab 5.1 (Biorad) was used to capture the gel image data.
Data analysis	Amber18 was used for molecular dynamics simulations and analyses. pymol (1.8) was used for visualization and analyses. Modeller 9.19 was used to model the mutant enzymes. Avogadro 1.1.1 was used to build the 3D structure of irone. MMPBSA.py software from Amber 18 suite is used to calculate MM/PBSA. R version 4.2.1 and microsoft excel 2016 were used to plot the graphs.

For manuscripts utilizing custom algorithms or software that are central to the research but not yet described in published literature, software must be made available to editors and reviewers. We strongly encourage code deposition in a community repository (e.g. GitHub). See the Nature Portfolio guidelines for submitting code & software for further information.

Data

Policy information about availability of data

All manuscripts must include a data availability statement. This statement should provide the following information, where applicable:

- Accession codes, unique identifiers, or web links for publicly available datasets
- A description of any restrictions on data availability
- For clinical datasets or third party data, please ensure that the statement adheres to our policy

All data supporting the findings of this study are available in the article and its supplementary files or upon request from the corresponding authors. The source data for all figures reported in the article and its supplementary information are provided in the Source Data file. The protein structure data generated in this article is

available in world wide Protein Data Bank under PDB ID 5GM1 (https://www.wwpdb.org/pdb?id=pdb_00005gm1), 5GM2 (https://www.wwpdb.org/pdb?id=pdb_00005gm2), 1KPG (https://www.wwpdb.org/pdb?id=pdb_00001KPG), 4F86 (https://www.wwpdb.org/pdb?id=pdb_00004F86), 4PNE (https://www.wwpdb.org/pdb?id=pdb_00004PNE) and Supplementary file 1. The initial and final configurations for molecular dynamics calculations are provided in Supplementary file 2. The sequence data, primers and gRNA for CRISPR-cas9 guided knockout used in this article are provided in the Supplementary file 3. Source data are provided with this paper.

Human research participants

Policy information about studies involving human research participants and Sex and Gender in Research.

Reporting on sex and gender	non applicable
Population characteristics	non applicable
Recruitment	non applicable
Ethics oversight	non applicable

Note that full information on the approval of the study protocol must also be provided in the manuscript.

Field-specific reporting

Please select the one below that is the best fit for your research. If you are not sure, read the appropriate sections before making your selection.

[x] Life sciences [ ] Behavioural & social sciences [ ] Ecological, evolutionary & environmental sciences

For a reference copy of the document with all sections, see nature.com/documents/nr-reporting-summary-flat.pdf

Life sciences study design

All studies must disclose on these points even when the disclosure is negative.

Sample size	The sample size calculation was not performed. As described in the figure legends, the cell lysate reactions (Fig. 2b and 3d) were performed with two biologically independent experiments (n=2), as the enzymes were grown and expressed from a large number of individual cells instead of from a single randomly picked colony. Purified enzymatic reactions are well controlled with defined components and concentrations. Thus, we reasoned that two or three biologically independent replicates are sufficient.
Data exclusions	No data were excluded.
Replication	All data has been repeated at least once to validate the results. All the data has been successfully reproduced.
Randomization	For in vivo production (Fig. 4), three colonies were randomly picked and grown up. For enzyme activity assay with different metabolites (Fig. 3a), master mixture of the enzyme reactions was randomly added into tubes containing different metabolites.
Blinding	No blinding was necessary, as the experiments were rationally designed with controls and no bias from the experimentalists.

Reporting for specific materials, systems and methods

We require information from authors about some types of materials, experimental systems and methods used in many studies. Here, indicate whether each material, system or method listed is relevant to your study. If you are not sure if a list item applies to your research, read the appropriate section before selecting a response.

Materials & experimental systems

n/a	Involved in the study	
[ ]	[x]	Antibodies
[x]	[ ]	Eukaryotic cell lines
[x]	[ ]	Palaeontology and archaeology
[x]	[ ]	Animals and other organisms
[x]	[ ]	Clinical data
[x]	[ ]	Dual use research of concern

Methods

n/a	Involved in the study	
[x]	[ ]	ChIP-seq
[x]	[ ]	Flow cytometry
[x]	[ ]	MRI-based neuroimaging

Antibodies

Antibodies used	HRP Anti-6X His tag® antibody [GT359] (ab184607) from abcam was used. As described in method section, it was diluted 2000 times in 1% milk in TBST buffer.
Validation	Purified his-tagged protein is used as a control to validate the antibody, as shown in Supplementary figure 3a. The antibody is HRP conjugated and species independent, so no further validation was performed.
